# Inducing a tolerogenic microenvironment within tissues

**DOI:** 10.1186/ar3406

**Published:** 2011-09-16

**Authors:** Stephen P Cobbold

**Affiliations:** 1 Sir William Dunn School of Pathology, University of Oxford, Oxford, OX1 3RE, UK

## 

The maintenance of tolerance to both self tissues foreign organ grafts depends on the activity of foxp3^+ ^regulatory T cells (Treg). We have used MHC-matched skin grafts as a model system to study how such Treg can be induced therapeutically and the mechanisms by which they act. Using monospecific TCR transgenic mice we have shown that a short treatment with monoclonal antibodies that block full T cell activation in vivo allows the targeted tissue to itself induce *de novo*, antigen specific, foxp3^+ ^Treg (iTreg) [1]. We also show that these iTreg are not only concentrated within the target tissue, but are continuously required to suppress the activity of primed effector cells also present within the tissue [2]. When taken together with previous findings of linked suppression and infectious tolerance [3], the evidence suggests that tolerance maintained by iTreg is dependent on a local, tolerogenic microenviroment within the tissue. One component of this microenvironment is the induction, by both innate inflammation and iTreg, of multiple enzymes that consume essential amino acids, including tryptophan, arginine and valine. Local amino acid depletion can be sensed by naïve and effector T cells, via the mammalian target of the immunosuppressive drug rapamycin (mTOR) pathway, which can synergise with TGFβ for the further induction of foxp3^+ ^iTreg [4]. TGFβ is also able to up-regulate the ectoenzymes CD39 and CD73 both on T cells and antigen presenting cells to catabolise inflammatory ATP to anti-inflammatory adenosine [5]. Microarray analysis of tolerated and control skin grafts for patterns of gene expression associated with the tolerogenic microenvironment confirms that these mechanisms are preferentially active locally within the tolerated tissues rather than throughout the systemic lymphoid system. Of particular interest, these same mechanisms seem to be active in grafted syngeneic tissues [6], suggesting that iTreg maintained microenvironments are important for maintaining self tolerance in the face of an inflammatory insult. The challenge now is how we can exploit appropriate combinations of T cell blockade, mTOR inhibition and TGFβ activation for translation to the clinic.
